# *KCNQ2* variants in neonatal onset of self-limiting epilepsy

**DOI:** 10.1515/crpm-2024-0039

**Published:** 2025-05-28

**Authors:** Shruthi Mundasad, Anthony R. Hart, Hannah K. Robinson, Anne Greenough

**Affiliations:** Department of Paediatric Neurology, King’s College Hospital NHS Foundation Trust, London, UK; Exeter Genomics Laboratory, Royal Devon University Healthcare NHS Foundation Trust, Exeter, UK; Department of Women and Children’s Health, School of Life Course Sciences, Faculty of Life Sciences and Medicine, Neonatal Intensive Care Centre, King’s College Hospital, King’s College London, London, UK

**Keywords:** neonatal self-limiting epilepsy, *KCNQ2*, genetic epilepsy

## Abstract

**Objectives:**

To describe the clinical presentation and response to medication in two cases of self-limiting *KCNQ2*-related epilepsy.

**Case presentation:**

Both infants were born at term and had tonic seizures during the first two weeks after birth. The first infant had frequent seizures at presentation requiring two weeks of hospital stay. The second infant was born three months later and was only briefly admitted to hospital. The first infant was conceived by sperm for *in vitro* fertilization donated by the second case’s father. Trio genome sequencing in case one successfully identified a pathogenic *KCNQ2* variant in the proband, which was also confirmed in the proband for case 2 by targeted Sanger sequencing. The second case’s father was an asymptomatic carrier of the pathogenic variant. Both infants responded to Carbamazepine. At more than six months of age, they are currently seizure free and developmentally normal.

**Conclusions:**

Self-limited epilepsies with onset in neonates (SeLNE) are usually autosomal dominant disorders characterized by the neonatal onset of focal motor seizures and the absence of neurodevelopmental complications. *KCNQ2*, encoding a voltage-gated potassium channel subunit, K_V_7.2, is the most common gene associated with SeLNE. Careful history taking and a genetic diagnosis can help to make the correct therapeutic choices.

## Introduction

Seizures are the most common neurological emergency in the newborn, occurring in between one and five per 1,000 live births [1]. The majority of seizures in the neonatal period occur in the context of an acute illness (“acute provoked” seizures, previously called “acute symptomatic”), but in some cases the seizures may be the first manifestation of early infantile epilepsy. The self-limited epilepsies with onset in neonates or infants (SeLNE), formerly called benign familial neonatal and/or infantile epilepsies, are autosomal dominant disorders and account for three percent of all cases of neonatal seizures. Over 80 % of SeLNE cases are due to pathogenic variants in the Potassium Voltage-Gated Channel Subfamily Q Member 2 (*KCNQ2*) gene and less commonly in *SCN2A* and *KCNQ3* [2].

Although seizures in infants with SeLNE often start between three and seven days after birth and become increasingly frequent, they subsequently remit spontaneously by around four to six months of age. About 90 % of patients with SeLNE have a normal neurodevelopment prognosis [1].

Sodium channel blocking anti-seizure medications, such as phenytoin, carbamazepine (CBZ), oxcarbazepine and lacosamide have been proven effective in neonates with channel-related epilepsy that may be refractory to other therapy [3], 4]. Approximately 80 % of *KCNQ2* and *SCN2A* related epilepsies have a full response to sodium channel blockers [5].

We report the clinical presentation and response to medication in two cases of self-limiting *KCNQ2*-related epilepsy.

## Case presentation

### Case 1

A first born female infant born to mothers in a same sex relationship via sperm donation. There was no family history of seizures in the delivering mother or the sperm donor. The delivering mother had spontaneous rupture of membranes for more than 24 h and received antibiotics. The infant was born at term by emergency caesarian section. The Apgar scores were 8 at 1 min and 10 at 5 min. The infant did not require antibiotics postnatally and was discharged home on day two after birth. The next day, the infant presented after two paroxysmal events. Following hospital admission, she had brief episodes of seizures, followed by prolonged seizures. Initially she was treated with a loading dose of phenobarbitone and intubated and ventilated. Amplitude integrated encephalography (aEEG) was commenced, and further clinical and electrical seizures were noted. The infant was given loading doses of phenytoin and levetiracetam and, the following day, she was started on a midazolam infusion. A maintenance dose of levetiracetam was commenced. By day three of admission, the seizures had ceased and neurological and systemic examination revealed no abnormalities. The infant had a raised CRP, CSF culture was negative, and the MRI were reported as unremarkable. The infant received antibiotics for two weeks as per the microbiology advice. The initial EEG showed non-specific findings, but the infant had breakthrough seizures on day 14. The EEG showed multifocal epileptiform discharges, more frequent over the left temporal region with relatively preserved background. Based on the presentation, clinical progress and investigations, a diagnosis of SeLNE was considered following a consultation with the paediatric neurologist, and the infant started on carbamazepine and genetic testing was ordered. The seizures immediately ceased and the infant was discharged home on day 25 of admission. Whole genome sequencing (WGS) testing revealed a heterozygous pathogenic *KCNQ2* variant in the infant and the sperm donor. Carbamazepine was weaned and stopped at six months. At nine months of age, the infant is seizure free and developmentally normal.

### Case 2

A first born girl born shortly after case one to the sperm donor of the first case. There was an unremarkable maternal history. The infant was born in good condition and was observed in the hospital for the first 24 h after birth. Physical and neurological examinations were normal. Safety net advice regarding the identification and first aid for seizures was given because of the paternal genetic result and the diagnosis of epilepsy in the first case. She was discharged home after 48 h. The parents noticed brief paroxysmal events at home during the first week and consulted the paediatric neurologist who knew them and the first case. Video footage showed suggested benign neonatal sleep myoclonus. During the second week of life, she developed episodes of limb stiffening, eye deviation to one side and reduced responsiveness. The parents and infant presented to the emergency department. EEG showed a focal abnormality suggestive of self limiting epilepsy and CT head was normal. Following consultation with the paediatric neurologist, the infant was treated with oral carbamazepine and the seizures immediately stopped. She was discharged home after two days. Targeted genetic testing identified the familial pathogenic *KCNQ2* variant.

Currently the infant is seven months old, is seizure free and developmentally normal and the carbamazepine has been stopped.

### Genetic analysis

For case one, rapid trio (proband and both biological parents) whole genome sequence analysis was performed [6]. Initially, a gene-agnostic trio analysis strategy, which uses inheritance-based variant filtering of ∼23,000 genes, was performed, which did not identify a clear likely genetic diagnosis. Notably, this strategy operates with the primary assumption that parents are unaffected and therefore does not retain single heterozygous variants present in the proband that have been inherited from a heterozygous parent. However, many early onset genetic epilepsies are often associated with reduced penetrance, i.e. not all individuals with a pathogenic variant are affected with the disorder. As such, further analysis of the genome sequencing data was performed to identify rare, heterozygous variants predicted deleterious in genes within the early onset or syndromic epilepsy v4.133 virtual gene panel (panelapp.genomicsengland.co.uk), which may have been inherited from an unaffected parent. This virtual gene panel analysis revealed heterozygosity for a *KCNQ2* nonsense variant, NM_172107.4: c.961C>T p.(Gln321Ter). The biological father was also found to be heterozygous for the variant. Genetic results were made available to the requesting clinical team within 14 days of sample receipt in the testing laboratory. The *KCNQ2* variant was absent from the Genome Aggregation Database (gnomAD) [7] and is predicted to introduce a premature termination codon, resulting in a transcript that is likely to be subject to nonsense-mediated decay, consistent with a haploinsufficiency mechanism. The variant was classified as pathogenic according to the ACMG standards and guidelines for the interpretation of sequence variants [8] and ACGS Best Practice Guidelines for Variant Classification in Rare Disease 2024 [9]. Targeted Sanger sequencing confirmed that case two was also heterozygous for the known familial pathogenic *KCNQ2* variant.

## Discussion

We report two cases with the same pathogenic nonsense variant in *KCNQ2* associated with a self-limited neonatal epilepsy phenotype. Both the cases experienced episodes of multifocal tonic seizures that responded to carbamazepine treatment.

The Potassium Voltage-Gated Channel Subfamily Q Member 2 (*KCNQ2*) gene is located on chromosome 20q13.33 and encodes the voltage-gated potassium channel subunit Kv7.2. The M channel is a heteromeric tetramer consisting of Kv7.2 and Kv7.3 subunits. When expressed individually, each subunit creates low-functioning channels. However, when both subunits are co-expressed, they generate functional channels that enhance the M-current by 10–50 times. This interaction would explain why pathogenic variants in either the *KCNQ2* or *KCNQ3* genes produce similar symptoms [10]. *In situ* hybridisation reveals that *KCNQ2* is predominantly found in the cerebellar cortex, the neocortex, and the hippocampal formation, including the dentate gyrus. This is noteworthy because these three structures display different susceptibility to epileptic seizures. *KCNQ3* is also localised to these areas in the mouse brain. However, the expression of *KCNQ2* occurs earlier than that of *KCNQ3* and increases rapidly during the first week of life. *KCNQ3* is expressed in very low amounts at birth, while *KCNQ2* is already expressed significantly [10]. This indicates that associations of *KCNQ2* and *KCNQ3* are likely to change as development progresses. The seizure remission is likely attributed to a compensation mechanism for *KCNQ2/KCNQ3* channel defects by other types of potassium channels that emerge after birth [11].

The phenotypes of *KCNQ2*-related disorders range from self-limited neonatal epilepsy to neurodevelopmental impairment or severe conditions, including developmental epileptic encephalopathy (DEE). Variability in phenotype may be associated with the location and type of variant. Other genetic or environmental factors may be involved in the disorder. Nearly 200 different variants have been identified in patients with SeLNE and DEE. SeLNE is thought to result from haploinsufficiency of *KCNQ2* and is predominantly caused by nonsense, frameshift and splicing variants, often inherited from a parent. On the other hand, *KCNQ2* variants associated with DEE are usually *de novo* and are often missense or in-frame insertions or deletions thought to act via a dominant-negative mechanism [3]. The clinical features indicating SeLNE include the absence of abnormality on brain MRI, normal interictal neurologic examination, normal early development, and remission of seizures by 12 months of age. On the other hand, the suspicion of *KCNQ2* DEE is based on the absence of structural lesions in the brain, severe abnormality on EEG background activity, poor response to epileptic medications, and developmental impairment [3].

The neurophysiological evaluation and monitoring by conventional EEG and aEEG (amplitude integrated encephalography) has become an integral part of clinical practice. The identification of a typical sequential seizure pattern with a prominent tonic onset on video-EEG in a neonate without structural brain damage or acute cause is a marker of genetic epilepsy due to *KCNQ2/KCNQ3/SCN2A* channelopathies. Most recent studies have found that patients with SeLNE typically have normal interictal EEG [4].

Various treatments, including sodium channel blockers (carbamazepine, oxcarbazepine, and phenytoin), and vitamin B6 have been found to treat seizures in SeLNE. When channelopathy is suspected as the cause of neonatal seizures, the ILAE (International League Against Epilepsy) task force recommends treatment with carbamazepine and phenytoin, with a high level of agreement among experts. However, there is insufficient evidence to recommend the use of any particular medication for the treatment of SeLNE. Further randomised controlled studies are needed to determine an optimal treatment [12], 13].

In case one, phenobarbital, phenytoin and levetiracetam were administered following local guidelines, but carbamazepine was commenced when a review of the case and the seizure semiology led to the diagnosis of SeLNE. In case two, carbamazepine treatment was started immediately because of the suspected likely diagnosis, and seizure control was achieved early, reducing the need for hospital stay.

## Conclusions

*KCNQ2* related self-limiting neonatal epilepsy is a genetic condition causing early onset seizures that typically resolve within a few months, with generally favourable prognosis and normal development. Rapid genome sequencing to identify the underlying genetic cause can aid prognostication, inform appropriate selection of anti-epileptic treatment and guide genetic counselling for the family, including recurrence risk in future offspring.

## Learning points

– Pathogenic variants in *KCNQ2* are responsible for around 80 % of cases of self-limited familial neonatal epilepsies, underscoring the importance of genetic testing.
– When self-limited familial epilepsy is suspected, antiseizure medication treatment with sodium channel blockers should be considered.
– Prognosis for self-limited familial epilepsy is favourable, with seizures typically disappearing between the first and twelfth month of life.
